# 1-Ammonio-1-phosphono­pentane-1-phospho­nic acid

**DOI:** 10.1107/S1600536808038968

**Published:** 2008-11-26

**Authors:** V. V. Bon, A. V. Dudko, A. N. Kozachkova, V. I. Pekhnyo

**Affiliations:** a V.I. Vernadskii Institute of General and Inorganic Chemistry, Kyiv 03680, Ukraine

## Abstract

The title compound, C_5_H_15_NO_6_P_2_, was obtained by the reaction of penta­nenitrile with PCl_3_ followed by the dropwise addition of water. The asymmetric unit contains one mol­ecule, which exists as a zwitterion with a positive charge on the –NH_3_ group and a negative charge on one of the phospho­nic O atoms. The crystal structure displays N—H⋯O and O—H⋯O hydrogen bonding, which creates a three-dimensional network.

## Related literature

For the biological activity of organic disphospho­nic acids, see: Matczak-Jon & Videnova-Adrabinska (2005 ▶); Szabo *et al.* (2002 ▶); Tromelin *et al.* (1986 ▶). For comparable bond lengths, see: Allen *et al.* (1987 ▶).
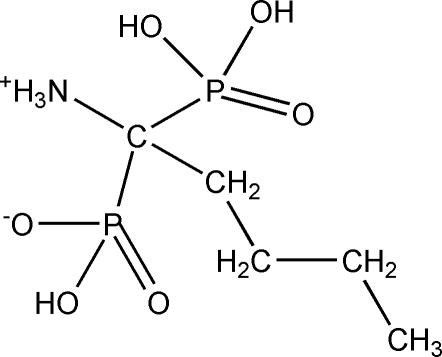


## Experimental

### 

#### Crystal data

C_5_H_15_NO_6_P_2_
*M* *_r_* = 247.12
Monoclinic, 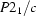

*a* = 14.5502 (3) Å
*b* = 7.1896 (1) Å
*c* = 9.4855 (2) Å
β = 96.938 (1)°
*V* = 985.01 (3) Å^3^
*Z* = 4
Mo *K*α radiation
μ = 0.45 mm^−1^
*T* = 100 (2) K
0.38 × 0.36 × 0.09 mm

#### Data collection

Bruker SMART APEXII CCD area-detector diffractometer
Absorption correction: multi-scan (*SADABS*; Bruker, 2005 ▶) *T*_min_ = 0.848, *T*_max_ = 0.961
23409 measured reflections
2471 independent reflections
2225 reflections with *I* > 2σ(*I*)
*R*_int_ = 0.043

#### Refinement

*R*[*F* ^2^ > 2σ(*F* ^2^)] = 0.028
*wR*(*F* ^2^) = 0.072
*S* = 1.06
2471 reflections
152 parameters
H atoms treated by a mixture of independent and constrained refinement
Δρ_max_ = 0.44 e Å^−3^
Δρ_min_ = −0.32 e Å^−3^

### 

Data collection: *APEX2* (Bruker, 2005 ▶); cell refinement: *SAINT* (Bruker, 2005 ▶); data reduction: *SAINT*; program(s) used to solve structure: *SHELXTL* (Sheldrick, 2008 ▶); program(s) used to refine structure: *SHELXTL*; molecular graphics: *SHELXTL*; software used to prepare material for publication: *SHELXTL*.

## Supplementary Material

Crystal structure: contains datablocks I, global. DOI: 10.1107/S1600536808038968/ez2151sup1.cif

Structure factors: contains datablocks I. DOI: 10.1107/S1600536808038968/ez2151Isup2.hkl

Additional supplementary materials:  crystallographic information; 3D view; checkCIF report

## Figures and Tables

**Table 1:** Hydrogen-bond geometry (Å, °)

*D*—H⋯*A*	*D*—H	H⋯*A*	*D*⋯*A*	*D*—H⋯*A*
N1—H1*N*⋯O4^i^	0.84 (2)	2.02 (2)	2.7584 (16)	146.7 (19)
N1—H2*N*⋯O5^ii^	0.91 (2)	1.88 (2)	2.7799 (16)	169.4 (19)
N1—H3*N*⋯O2^iii^	0.88 (2)	1.99 (2)	2.8451 (16)	162.9 (18)
O1—H1*O*⋯O2^iii^	0.79 (2)	1.85 (3)	2.6297 (15)	167 (3)
O3—H3*O*⋯O5^iii^	0.79 (3)	1.70 (3)	2.4884 (15)	175 (3)
O6—H6*O*⋯O4^iv^	0.83 (3)	1.72 (3)	2.5372 (14)	168 (3)

Symmetry codes: (i) 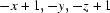; (ii) 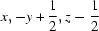; (iii) 
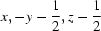
; (iv) 
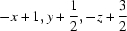
.

**Fig. 1.**
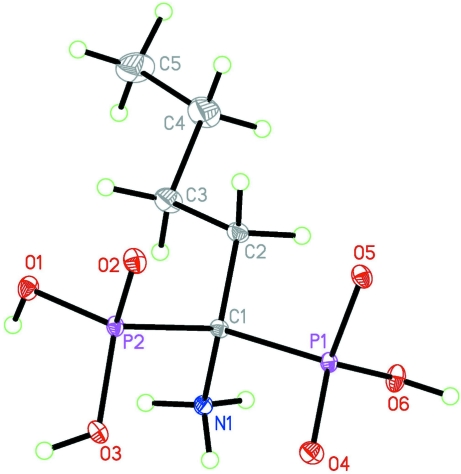
The asymmetric unit of the title compound showing the atom-labelling scheme and 50% probability displacement ellipsoids for the non-hydrogen atoms.

**Fig. 2.**
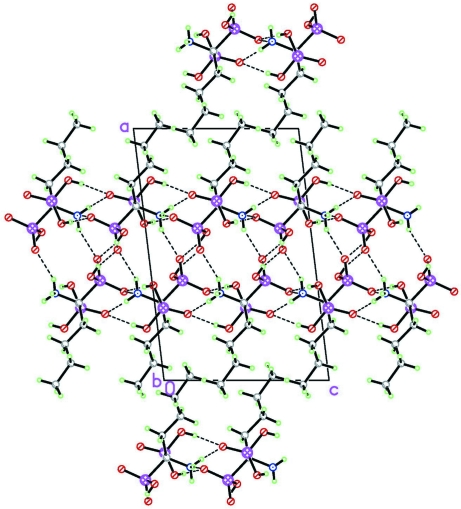
Crystal packing of the title compound; projection along b axis. Dashed lines indicate hydrogen bonds.

## supplementary crystallographic information

### Comment

Organic diphosphonic acids are potentially very powerful chelating agents used in metal extractions and have been tested by the pharmaceutical industry for use as efficient drugs preventing calcification and inhibiting bone resorption (Tromelin *et al.*, 1986, Matczak-Jon & Videnova-Adrabinska, 2005). Diphosphonic acids are used in the treatment of Paget disease, osteoporosis and tumoral osteolysis (Szabo *et al.*, 2002).

The asymmetric unit of the title compound contains one molecule, which exists as a zwitterion with positive and negative charges on the NH_3_ group and one of the phosphonic oxygen atoms, respectively. The phosphorus atoms display slightly distorted tetrahedral geometries, each provided by three oxygen atoms and one carbon atom. Bond lengths and angles have normal values (Allen *et al.*, 1987).

There are no solvent water molecules in the asymmetric unit, which is unusual for α-aminodiphosphonic acids. This fact can be explained by the presence of the hydrophobic alkyl group. The structure is stabilized by a three-dimensional O—H···O and N—H···O hydrogen bonding network (Fig. 1, Table 1).

### Experimental

Dry hydrogen chloride at about 278 K was brought into contact with the surface of a mixture of pentanenitrile (83.13 g, 1 mol) and PCl_3_ (87.4 ml, 1 mol). After an hour water (54 ml, 3 mol) was added to the mixture dropwise. After a day the solution was treated by an excess amount of water and then vacuum distilled. The obtained solution was treated by a mixture of acetone and diethyl ether, yielding colourless crystals of the title compound.

### Refinement

H atoms bonded to O and N atoms were located in a difference map. Other H atoms bonded to C atoms were positioned geometrically and refined using a riding model with C—H = 0.98 Å for CH_3_ [*U*_iso_(H) = 1.5Ueq(C)] and C—H = 0.99 Å for CH_2_ [*U*_iso_(H) = 1.2Ueq(C)]

### Crystal data

**Table:**

C_5_H_15_NO_6_P_2_	*F*_000_ = 520
*M**_r_* = 247.12	*D*_x_ = 1.666 Mg m^−^^3^
Monoclinic, *P*2_1_/*c*	Melting point: 562 K
Hall symbol: -P 2ybc	Mo *K*α radiation λ = 0.71073 Å
*a* = 14.5502 (3) Å	Cell parameters from 8806 reflections
*b* = 7.1896 (1) Å	θ = 2.8–28.4º
*c* = 9.4855 (2) Å	µ = 0.45 mm^−^^1^
β = 96.938 (1)º	*T* = 100 (2) K
*V* = 985.01 (3) Å^3^	Plate, colourless
*Z* = 4	0.38 × 0.36 × 0.09 mm

### Data collection

**Table:**

Bruker SMART APEXII CCD area-detector diffractometer	2471 independent reflections
Radiation source: fine-focus sealed tube	2225 reflections with *I* > 2σ(*I*)
Monochromator: graphite	*R*_int_ = 0.043
*T* = 100(2) K	θ_max_ = 28.4º
φ and ω scans	θ_min_ = 2.8º
Absorption correction: multi-scan(SADABS; Bruker, 2005)	*h* = −19→19
*T*_min_ = 0.848, *T*_max_ = 0.961	*k* = −9→9
23409 measured reflections	*l* = −12→12

### Refinement

**Table:**

Refinement on *F*^2^	Secondary atom site location: difference Fourier map
Least-squares matrix: full	Hydrogen site location: inferred from neighbouring sites
*R*[*F*^2^ > 2σ(*F*^2^)] = 0.028	H atoms treated by a mixture of independent and constrained refinement
*wR*(*F*^2^) = 0.072	*w* = 1/[σ^2^(*F*_o_^2^) + (0.0317*P*)^2^ + 0.8863*P*] where *P* = (*F*_o_^2^ + 2*F*_c_^2^)/3
*S* = 1.06	(Δ/σ)_max_ < 0.001
2471 reflections	Δρ_max_ = 0.44 e Å^−^^3^
152 parameters	Δρ_min_ = −0.32 e Å^−^^3^
Primary atom site location: structure-invariant direct methods	Extinction correction: none

### Special details

**Table:**

Geometry. All e.s.d.'s (except the e.s.d. in the dihedral angle between two l.s. planes) are estimated using the full covariance matrix. The cell e.s.d.'s are taken into account individually in the estimation of e.s.d.'s in distances, angles and torsion angles; correlations between e.s.d.'s in cell parameters are only used when they are defined by crystal symmetry. An approximate (isotropic) treatment of cell e.s.d.'s is used for estimating e.s.d.'s involving l.s. planes.
Refinement. Refinement of *F*^2^ against ALL reflections. The weighted *R*-factor *wR* and goodness of fit *S* are based on *F*^2^, conventional *R*-factors *R* are based on *F*, with *F* set to zero for negative *F*^2^. The threshold expression of *F*^2^ > σ(*F*^2^) is used only for calculating *R*-factors(gt) *etc*. and is not relevant to the choice of reflections for refinement. *R*-factors based on *F*^2^ are statistically about twice as large as those based on *F*, and *R*- factors based on ALL data will be even larger.

### Fractional atomic coordinates and isotropic or equivalent isotropic displacement parameters (Å^2^)

**Table:**

	*x*	*y*	*z*	*U*_iso_*/*U*_eq_	
P1	0.39648 (2)	0.07886 (5)	0.68586 (4)	0.00647 (9)	
P2	0.28775 (2)	−0.24846 (5)	0.54892 (4)	0.00680 (9)	
C1	0.30732 (9)	0.00397 (19)	0.54146 (14)	0.0067 (3)	
C2	0.21901 (10)	0.1205 (2)	0.55184 (15)	0.0100 (3)	
H2A	0.1914	0.0778	0.6366	0.012*	
H2B	0.2383	0.2514	0.5692	0.012*	
C3	0.14290 (10)	0.1183 (2)	0.42611 (16)	0.0139 (3)	
H3A	0.1698	0.1489	0.3380	0.017*	
H3B	0.1157	−0.0079	0.4153	0.017*	
C4	0.06761 (12)	0.2576 (3)	0.4478 (2)	0.0239 (4)	
H4A	0.0485	0.2381	0.5434	0.029*	
H4B	0.0939	0.3844	0.4457	0.029*	
C5	−0.01700 (12)	0.2475 (3)	0.3401 (2)	0.0238 (4)	
H5A	0.0005	0.2714	0.2452	0.036*	
H5B	−0.0620	0.3411	0.3624	0.036*	
H5C	−0.0447	0.1234	0.3424	0.036*	
N1	0.34540 (9)	0.04748 (18)	0.40433 (12)	0.0075 (2)	
O1	0.20552 (7)	−0.29755 (15)	0.43309 (11)	0.0103 (2)	
O2	0.26347 (7)	−0.29945 (15)	0.69151 (10)	0.0105 (2)	
O3	0.37688 (7)	−0.33350 (15)	0.50568 (11)	0.0102 (2)	
O4	0.48097 (7)	−0.03933 (15)	0.68442 (11)	0.0106 (2)	
O5	0.35286 (7)	0.08211 (14)	0.82242 (10)	0.0092 (2)	
O6	0.41170 (7)	0.28091 (15)	0.63361 (11)	0.0103 (2)	
H1N	0.4006 (14)	0.015 (3)	0.406 (2)	0.015 (5)*	
H2N	0.3439 (14)	0.172 (3)	0.387 (2)	0.022 (5)*	
H3N	0.3141 (13)	−0.009 (3)	0.331 (2)	0.014 (5)*	
H1O	0.2192 (17)	−0.283 (4)	0.356 (3)	0.032 (6)*	
H3O	0.3713 (18)	−0.416 (4)	0.450 (3)	0.040 (7)*	
H6O	0.4518 (18)	0.340 (4)	0.684 (3)	0.046 (8)*	

### Atomic displacement parameters (Å^2^)

**Table:**

	*U*^11^	*U*^22^	*U*^33^	*U*^12^	*U*^13^	*U*^23^
P1	0.00764 (16)	0.00625 (17)	0.00532 (16)	−0.00016 (12)	−0.00004 (12)	−0.00001 (12)
P2	0.00865 (17)	0.00661 (17)	0.00509 (16)	−0.00071 (12)	0.00064 (12)	−0.00014 (12)
C1	0.0075 (6)	0.0081 (6)	0.0045 (6)	0.0003 (5)	0.0007 (5)	0.0000 (5)
C2	0.0085 (6)	0.0107 (7)	0.0107 (6)	0.0022 (5)	0.0010 (5)	−0.0017 (5)
C3	0.0119 (7)	0.0176 (8)	0.0114 (7)	0.0050 (6)	−0.0014 (5)	−0.0005 (6)
C4	0.0146 (8)	0.0292 (10)	0.0264 (9)	0.0094 (7)	−0.0043 (7)	−0.0081 (7)
C5	0.0140 (7)	0.0316 (10)	0.0249 (9)	0.0078 (7)	−0.0024 (6)	0.0026 (7)
N1	0.0082 (6)	0.0087 (6)	0.0058 (5)	−0.0007 (4)	0.0011 (4)	−0.0002 (4)
O1	0.0113 (5)	0.0123 (5)	0.0070 (5)	−0.0025 (4)	0.0002 (4)	−0.0008 (4)
O2	0.0142 (5)	0.0105 (5)	0.0068 (4)	−0.0006 (4)	0.0018 (4)	0.0006 (4)
O3	0.0116 (5)	0.0083 (5)	0.0109 (5)	0.0009 (4)	0.0022 (4)	−0.0026 (4)
O4	0.0095 (5)	0.0122 (5)	0.0096 (5)	0.0026 (4)	−0.0004 (4)	−0.0001 (4)
O5	0.0123 (5)	0.0087 (5)	0.0067 (4)	−0.0001 (4)	0.0016 (4)	−0.0003 (4)
O6	0.0134 (5)	0.0079 (5)	0.0089 (5)	−0.0031 (4)	−0.0007 (4)	0.0013 (4)

### Geometric parameters (Å, °)

**Table:**

P1—O4	1.4959 (10)	C3—H3A	0.9900
P1—O5	1.5099 (10)	C3—H3B	0.9900
P1—O6	1.5593 (11)	C4—C5	1.504 (2)
P1—C1	1.8492 (14)	C4—H4A	0.9900
P2—O2	1.4846 (10)	C4—H4B	0.9900
P2—O3	1.5337 (11)	C5—H5A	0.9800
P2—O1	1.5639 (10)	C5—H5B	0.9800
P2—C1	1.8398 (14)	C5—H5C	0.9800
C1—N1	1.5070 (17)	N1—H1N	0.84 (2)
C1—C2	1.5471 (19)	N1—H2N	0.91 (2)
C2—C3	1.5264 (19)	N1—H3N	0.88 (2)
C2—H2A	0.9900	O1—H1O	0.79 (2)
C2—H2B	0.9900	O3—H3O	0.79 (3)
C3—C4	1.517 (2)	O6—H6O	0.83 (3)
			
O4—P1—O5	116.59 (6)	C2—C3—H3A	109.5
O4—P1—O6	112.20 (6)	C4—C3—H3B	109.5
O5—P1—O6	110.42 (6)	C2—C3—H3B	109.5
O4—P1—C1	109.37 (6)	H3A—C3—H3B	108.1
O5—P1—C1	108.06 (6)	C5—C4—C3	114.90 (15)
O6—P1—C1	98.61 (6)	C5—C4—H4A	108.5
O2—P2—O3	116.57 (6)	C3—C4—H4A	108.5
O2—P2—O1	109.77 (6)	C5—C4—H4B	108.5
O3—P2—O1	108.78 (6)	C3—C4—H4B	108.5
O2—P2—C1	109.49 (6)	H4A—C4—H4B	107.5
O3—P2—C1	104.08 (6)	C4—C5—H5A	109.5
O1—P2—C1	107.72 (6)	C4—C5—H5B	109.5
N1—C1—C2	109.71 (11)	H5A—C5—H5B	109.5
N1—C1—P2	108.22 (9)	C4—C5—H5C	109.5
C2—C1—P2	113.48 (10)	H5A—C5—H5C	109.5
N1—C1—P1	106.30 (9)	H5B—C5—H5C	109.5
C2—C1—P1	107.99 (9)	C1—N1—H1N	112.5 (13)
P2—C1—P1	110.90 (7)	C1—N1—H2N	111.0 (13)
C3—C2—C1	118.31 (12)	H1N—N1—H2N	106.4 (19)
C3—C2—H2A	107.7	C1—N1—H3N	112.3 (12)
C1—C2—H2A	107.7	H1N—N1—H3N	106.5 (18)
C3—C2—H2B	107.7	H2N—N1—H3N	107.9 (18)
C1—C2—H2B	107.7	P2—O1—H1O	111.4 (17)
H2A—C2—H2B	107.1	P2—O3—H3O	117.1 (19)
C4—C3—C2	110.76 (13)	P1—O6—H6O	114.5 (19)
C4—C3—H3A	109.5		
			
O2—P2—C1—N1	171.72 (8)	O4—P1—C1—C2	176.54 (9)
O3—P2—C1—N1	46.42 (10)	O5—P1—C1—C2	48.68 (11)
O1—P2—C1—N1	−68.96 (10)	O6—P1—C1—C2	−66.19 (10)
O2—P2—C1—C2	−66.27 (11)	O4—P1—C1—P2	51.62 (9)
O3—P2—C1—C2	168.44 (9)	O5—P1—C1—P2	−76.24 (8)
O1—P2—C1—C2	53.05 (11)	O6—P1—C1—P2	168.90 (7)
O2—P2—C1—P1	55.49 (9)	N1—C1—C2—C3	50.75 (17)
O3—P2—C1—P1	−69.81 (8)	P2—C1—C2—C3	−70.43 (15)
O1—P2—C1—P1	174.81 (7)	P1—C1—C2—C3	166.21 (11)
O4—P1—C1—N1	−65.79 (10)	C1—C2—C3—C4	−173.11 (14)
O5—P1—C1—N1	166.35 (9)	C2—C3—C4—C5	−171.76 (15)
O6—P1—C1—N1	51.49 (10)		

### Hydrogen-bond geometry (Å, °)

**Table:**

*D*—H···*A*	*D*—H	H···*A*	*D*···*A*	*D*—H···*A*
N1—H1N···O4^i^	0.84 (2)	2.02 (2)	2.7584 (16)	146.7 (19)
N1—H2N···O5^ii^	0.91 (2)	1.88 (2)	2.7799 (16)	169.4 (19)
N1—H3N···O2^iii^	0.88 (2)	1.99 (2)	2.8451 (16)	162.9 (18)
O1—H1O···O2^iii^	0.79 (2)	1.85 (3)	2.6297 (15)	167 (3)
O3—H3O···O5^iii^	0.79 (3)	1.70 (3)	2.4884 (15)	175 (3)
O6—H6O···O4^iv^	0.83 (3)	1.72 (3)	2.5372 (14)	168 (3)

Symmetry codes: (i) −*x*+1, −*y*, −*z*+1; (ii) *x*, −*y*+1/2, *z*−1/2; (iii) *x*, −*y*−1/2, *z*−1/2; (iv) −*x*+1, *y*+1/2, −*z*+3/2.
